# Comparative Study of Protective Effect of Cimetidine and Verapamil on Paracetamol-Induced Hepatotoxicity in Mice

**DOI:** 10.1155/2020/9185361

**Published:** 2020-01-23

**Authors:** Lubna Danish, Riffat Siddiq, Sarwat Jahan, Mehwish Taneez, Manzoor Khan, Marva Sandhu

**Affiliations:** ^1^Sulaiman Bin Abdullah Aba Al-Khail Centre for Interdisciplinary Research in Basic Sciences (CIRBS), International Islamic University Islamabad (IIUI), Islamabad, Pakistan; ^2^Department of Maxillofacial Surgery, Rehman College of Dentistry, Peshawar, Pakistan; ^3^Northwest School of Medicine, Phase-V Hayatabad, Peshawar, Pakistan; ^4^Department of Medicine, Khyber Teaching Hospital, Peshawar, Pakistan; ^5^Department of Pharmacology, National Institute of Health (NIH), Islamabad, Pakistan

## Abstract

Paracetamol, chemically known as acetaminophen, if taken in higher doses has hepatotoxic potential. Cimetidine by inhibiting the cytochromal enzymes and reducing the production of the toxic metabolite can reduce the hepatotoxic potential while Verapamil can act as a hepatoprotective by maintaining calcium homeostasis. The present study was conducted to study the hepatoprotective activity of Cimetidine and Verapamil against the toxicity induced by paracetamol. In addition to the group receiving only distilled water or 300 mg/kg paracetamol additional groups were added treated with 150 mg/kg Cimetidine and Verapamil alone or both. The Liver function tests and histopathology revealed hepatotoxicity in the group receiving paracetamol (PCM) while normal parameters were observed in the groups receiving Cimetidine and Verapamil. Our results strongly suggested that Cimetidine and Verapamil possess hepatoprotective potential against paracetamol induced hepatotoxicity.

## 1. Introduction

Damage to living tissue will result in a response known as inflammation. The main purpose of inflammation is to localize the agent causing damage and to remove it as soon as possible; it will lead to healing of the body [1]. One of the important mediators in case of inflammation is prostaglandin and inhibition of this mediator is an important target for treatment [2]. Drugs most commonly used in case of acute and chronic inflammation are: Naproxen, Ibuprofen, Aspirin, Acetaminophen, Corticosteroids etc. Paracetamol chemically known as acetaminophen was first described as an analgesic and antipyretic in 1893 by Von Mering [3]. Clear mechanism of paracetamol is still not known and it is still debatable however most widely it is accepted that the primary site of its action is inhibiting prostaglandins production or action on cannabinoid receptors through an active metabolite [4]. Two main factors on which paracetamol metabolism depends is the dose of the drug and age of the patient [5]. In healthy adult acetaminophen is metabolizes into glucuronide, sulphate, and cysteine metabolites. Paracetamol is a relatively safe drug with very low propensity to produce adverse reactions when it is ingested in therapeutic doses, but if taken in higher doses it has hepatotoxic potential [6]. Metabolism of paracetamol to a nontoxic metabolite takes place, primarily through the process of conjugation in the liver [7]. In cases of acute over dosage of this drug, there is saturation of conjugation which leads to excess production of Acetyl para amino phenol (APAP). APAP then undergoes oxidative metabolism through cytochromal enzymes into a toxic metabolite damaging the liver [8].

Cimetidine is included in a group of drugs known as H2 blockers. Cimetidine is one of the enzyme inducer drugs i.e., it decreases the activity of the cytochromal enzymes [9]. Cimetidine has the property of inhibiting the cytochromal enzyme CYP3A4 [10], enzyme CYP2D6 [11], and enzyme CYP1A2 [12]. Toxic metabolite are produced as a result of metabolism of paracetamol by the cytochromal enzymes is inhibited by cimetidine. It also reduces both the rate as well as the degree to which glutathione stores are depleted by paracetamol.

The second drug Verapamil, a calcium channel blocker, is added to study its hepatoprotective activity against paracetamol. An increased level of cytosolic calcium is observed by the toxic dose of paracetamol in an isolated hepatocyte model by Li and colleagues [13]. Prevention of this rise in calcium will lead to the protection of hepatic damage, so the calcium level can be best controlled with the help of a Ca antagonist. Role of calcium ion in the destruction of liver cells is an important factor, which can be prevented with the help of calcium channel blocker (Verapamil) along with other inducers the amount of calcium release will affect the mitochondrial membrane permeability transition (MPT). The MPT is Ca-dependent and can be inhibited by blocking Ca influx [14]. The permeability of mitochondrial membrane increases along with inhibition of the plasma membrane for calcium. The ultimate result will be increase in cytosolic Ca level, which is a trigger to the production of proteases. The production of proteases is calcium dependent. Inhibition of calcium channel at this stage will inhibit the production of enzymes and destruction of liver cell can be protected. Destruction of the nucleus is also calcium dependent production of endonuclease. Protection of calcium levels with the help of calcium channel blocker will prevent nucleus and DNA fragmentation [15].

This study is designed to understand and compare the protective effect of Cimetidine and Verapamil on the hepatotoxicity induced by paracetamol.

## 2. Materials and Methods

### 2.1. Study Settings

This study was a laboratory based randomized controlled trial. The study was carried out in the Veterinary Research Institute (VRI), Peshawar in collaboration with the Pharmacology Department of Khyber Girls Medical College (KGMC) and Khyber Medical University (KMU), Peshawar. The study is approved by the ethics committee of the Veterinary Research Institute (VRI), Peshawar and Pharmacology, Department of Khyber Girls Medical College. Male mice, 25–30 grams of weight were placed for a week in the animal house of VRI Peshawar under standard conditions of temperature between 20 and 25°C. Mice were given proper diet and water throughout the duration of the study.

### 2.2. Sample Size and Collection

BALB/C mice were initially selected through a nonprobability convenience sampling method. A total of 50 mice of BALB/C type were randomly sorted into a total of 5 groups; each of the groups had 10 animals (Table 1). Mice having a prominent deformity as well as inactive mice were excluded from the study. All the animals were sacrificed on day 14. Terminal cardiac blood samples were taken for the assessment of the liver enzymes, Bilirubin, Alkaline Phosphatase (ALP), Aspartate Aminotransferase (ALT), and Alanine Aminotransferase. The liver was dissected out after which its preservation was done in 10% of formalin solution. Tissues were processed to divide the liver into various sections. Hematoxylin and Eosin (H & E) were used for staining.

### 2.3. Chemicals and Drugs

Paracetamol (300 mg/kg) was injected intraperitoneally; 150 mg/kg of each Cimetidine and Verapamil dose was used. All drugs were purchased from Sigma-Aldrich (USA). The tissues were preserved in 10% formaldehyde. Chloroform was used for anesthetizing the animals.

### 2.4. Generation of Paracetamol Induced Hepatotoxic Model

The hepatotoxic dose of paracetamol and hepatoprotective doses of Verapamil and Cimetidine were confirmed after the conduction of a pilot study conducted on a total of 10 male BALB/C mice. A dose of 300 mg/kg of paracetamol, 150 mg/kg of Verapamil and 150 mg/kg of Cimetidine with the time interval of 24 hours for 2 weeks was given. At the end of 2 weeks terminal sampling and dissection was selected on the basis of previous findings.

### 2.5. Blood Sampling and Tissue Collection

Terminal blood sampling was performed through cardiac puncture for all the groups at the end of two weeks, 24 hours after the last dose of the drugs. Samples were taken to the Clinical Pathology Laboratory where they were centrifuged as to separate the serum for the assessment of Liver function tests. For tissue extraction the anesthetized mouse was fixed on the dissection board, the liver was pushed out of the abdominal cavity. After washing off excess blood, it was immediately fixed in 10% formalin. Serial dehydration was done by passing the tissue through increasing concentrations of alcohol (70%, 80%, and 90%). Following clearing with xylene, the tissues were impregnated with paraffin in an incubator at 58–60°C for an hour. After embedding tissues in blocks, sections with H & E staining were examined thoroughly by light microscopy. Knodell Modified scoring system was used for grading the histopathological changes in liver.

### 2.6. Determination of Hepatic Dysfunction

Dysfuntioning of Liver was estimated by measuring Alanine aminotransferase (ALT), Alkaline-Phosphatase (ALP), Aspartate Aminotransferase (AST), and Bilirubin. Commercially available kits were used for the assessment of serum levels of the respective enzyme's activity.

### 2.7. Statistical Analysis

The expression of the obtained results was done in the form of mean value as well as standard deviation. The statistics were calculated on the statistical package used for social sciences version 23. Comparison of biochemical markers at initial and final hours in the same group was calculated with one way ANOVA. It was then followed by another test called Post Hoc Tukey. *p* < 0.05 was taken as significant value.

## 3. Results

### 3.1. Biochemical and Histological Analysis of Different Group

#### 3.1.1. Group 1 (Control Group)

**Figure 1:**
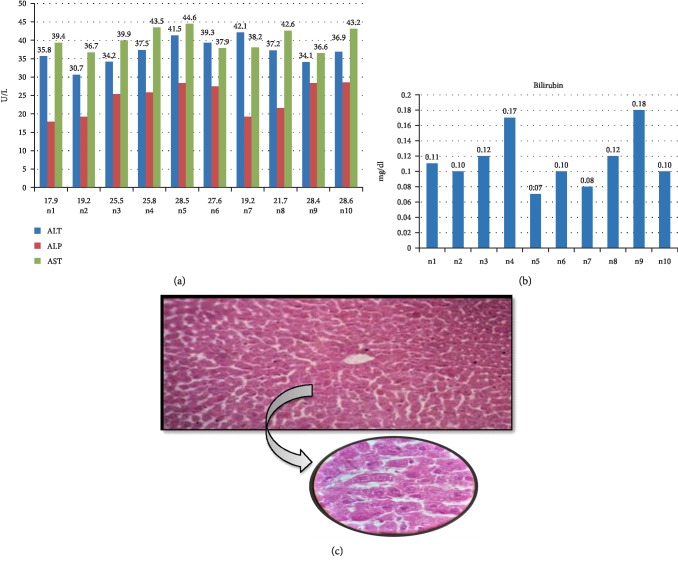
Biochemical and histological analysis of group 1. (a) ALT, ALP, and AST serum content in control group. Mice were given either saline or distilled water for two weeks. *X*-axis represents the mice while *Y*-axis shows the serum content of liver function enzymes. (b) Bilirubin content of control mice after two weeks treated with only saline or distilled water. *Y*-axis shows the serum level of Bilirubin (mg/dl) while *X*-axis represents the number of mice. (c) Histological examination of liver tissues of control group. After treatment with saline and distilled water for two weeks, liver tissues with fixed in 10% formaldehyde, and proceed with H & E staining as described in Section 2.

Elevated serum levels of liver function markers is the first sign of hepatotoxicity, we confirmed the paracetamol-induced liver toxicity by measuring serum content of ALT, ALP, AST, and Bilirubin. Group 1 was divided into two subgroups. Group 1a received intraperitoneal normal saline while Group 1b received intraperitoneal distilled water daily for 2 weeks. The liver parameters remained with normal limits where serum ALT level was 36.93 ± 3.48 U/L, ALP had a mean value of 24.24 ± 4.31 U/L, Serum AST had a mean value of 40.26 ± 2.98 U/L, and Bilirubin showed a mean value of 0.11 ± 0.03 mg/dl. Histopathology of group I coincided with the biochemical parameters, graded as normal according to ISHAK's criteria (Figure 1).

#### 3.1.2. Group 2 (PMC Group)

**Figure 2:**
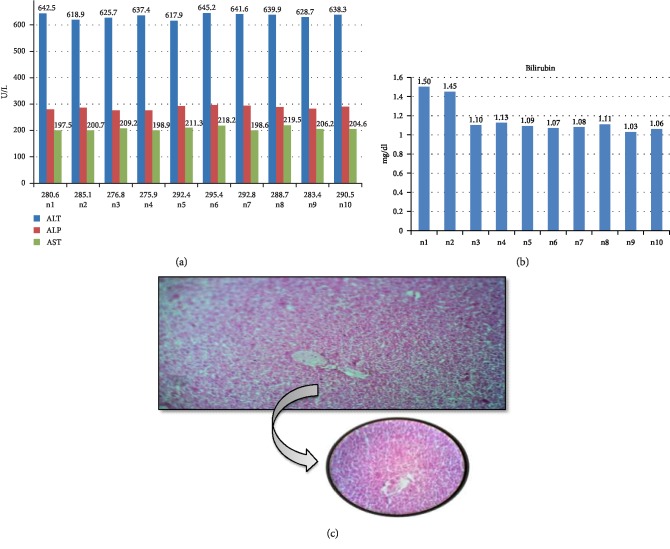
Biochemical and histological analysis of group 2. (a) ALT, ALP, and AST serum content in group 2. Mice were given 300 mg per kg of paracetamol via intraperitoneal injection for two weeks. *X*-axis represents the mice while *Y*-axis shows the serum content of liver function enzymes. (b) Bilirubin content of group 2 (PMC group) mice after two weeks treated with 300 mg/kg paracetamol. *Y*-axis shows the serum level of Bilirubin (mg/dl) while *X*-axis represents the number of mice. (c) Histological examination of liver tissues of PMC group. After treatment with 300 mg/kg paracetamol for two weeks, liver tissues were fixed in 10% formaldehyde, and proceed with H & E staining as described in Section 2.

To assess the toxicity induced by paracetamol, the serum level of different liver enzymes (ALT, AST, and ALP) were measured. Group 2 received paracetamol through I/P injection daily. After 2 weeks there was a significant rise in all the liver function tests with mean serum ALT having a mean value of 633.61 ± 10.01 U/L, serum ALP levels with a mean value of 286.16 ± 6.88 U/L, AST levels showed a mean value of 206.47 ± 7.99 U/L, and serum Bilirubin had a mean value of 1.16 ± 0.16 mg/dl. Light microscopy of slides classed slides as moderate damage (Figure 2).

#### 3.1.3. Group 3 (PCM/Verapamil Group)

**Figure 3:**
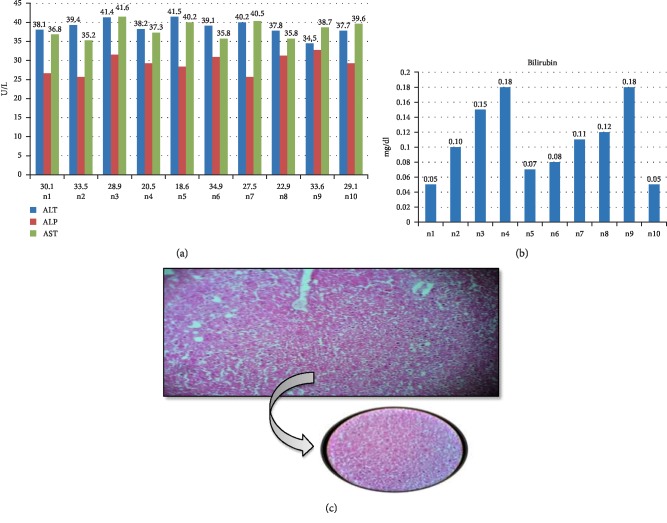
Biochemical and histological analysis of group 3. (a) ALT, ALP, and AST serum content in group 3 (PCM/Verapamil group). Mice were given 300 mg per kg of paracetamol and 150 mg per kg Cimetidine via intraperitoneal injection for two weeks. *X*-axis represents the mice while *Y*-axis shows the serum content of liver function enzymes. (b) Bilirubin content of group 3 mice after two weeks treated with 300 mg/kg paracetamol plus 150 mg/kg Cimetidine. *Y*-axis shows the serum level of Bilirubin (mg/dl) while *X*-axis represents the number of mice. (c) Histological examination of liver tissues of PMC Cimetidine group. After treatment with 300 mg/kg paracetamol and 150 mg/kg Cimetidine for two weeks, liver tissues with fixed in 10% formaldehyde, and proceed with H & E staining as described in Section 2.

The Group 3 was given daily I/P paracetamol in normal saline and I/P Cimetidine in distilled water daily for 2 weeks. The liver function tests revealed normal parameter with mean serum ALT of 38.79 ± 2.05 U/L, mean serum ALP of 29.18 ± 2.49 U/L, mean serum AST of 38.16 ± 2.26 U/L, and mean serum Bilirubin of 0.10 ± 0.04 mg/dl. In this group, after light microscopy of H & E stained slides were classed to have minimal histopathological changes (Figure 3).

#### 3.1.4. Group 4 (PCM/Cimetidine Group)

**Figure 4:**
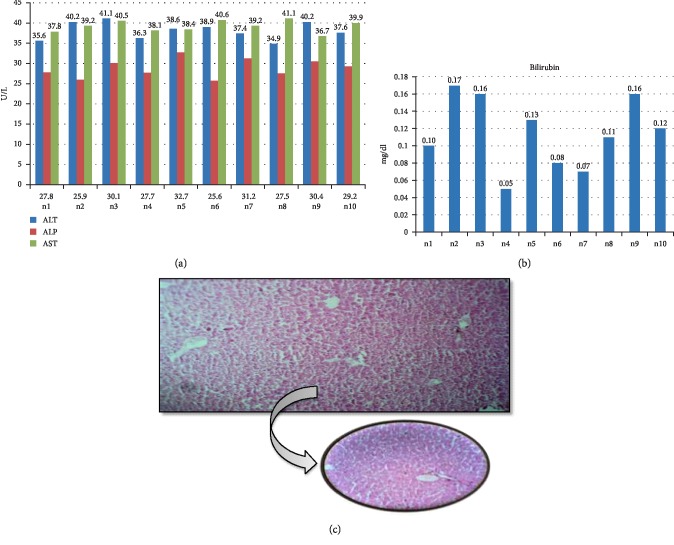
Biochemical and histological analysis of group 4. (a) ALT, ALP and AST serum content in group 4 (PCM/Cimetidine group). Mice were given 300 mg per kg of paracetamol and 150 mg per kg Cimetidine via intraperitoneal injection for two weeks. *X*-axis represents the mice while *Y*-axis shows the serum content of liver function enzymes. (b) Bilirubin content of group 4 mice after two weeks treated with 300 mg/kg paracetamol plus 150 mg/kg Cimetidine. *Y*-axis shows the serum level of Bilirubin (mg/dl) while *X*-axis represents the number of mice. (c) Histological examination of liver tissues of PMC/Cimetidine group. After treatment with 300 mg/kg paracetamol and 150 mg/kg Cimetidine for two weeks, liver tissues with fixed with 10% formaldehyde, and proceed with H & E staining as described in Section 2.

The Group 4 was given daily I/P paracetamol in normal saline and I/P Cimetidine in normal saline daily for 2 weeks. The liver function tests revealed normal parameter with mean serum ALT of 38.79 ± 2.05 U/L, mean serum ALP of 28.81 ± 2.30 U/L, mean serum AST of 39.15 ± 1.40 U/L and mean serum Bilirubin of 0.11 ± 0.04 mg/dl. In this group, light microscopy of H & E stained slides was classed to have minimal histopathological changes (Figure 4).

#### 3.1.5. Group 5 (PCM/Verapamil/Cimetidine Group)

**Figure 5:**
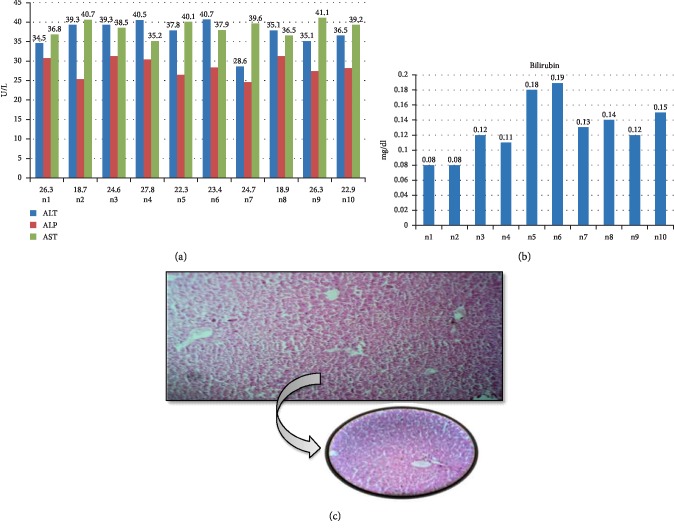
Biochemical and histological analysis of group 5. (a) ALT, ALP and AST serum content in group 5 (PCM/Verapamil/Cimetidine group). Mice were given 300 mg per kg of paracetamol, 150 mg/kg Verapamil and 150 mg per kg Cimetidine via intraperitoneal injection for two weeks. *X*-axis represents the mice while *Y*-axis shows the serum content of liver function enzymes. (b) Bilirubin content of group 5 mice after two weeks treated with 300 mg/kg paracetamol, 150 mg/kg Verapamil and 150 mg/kg Cimetidine. *Y*-axis shows the serum level of Bilirubin (mg/dl) while *X*-axis represents the number of mice. (c) Histological examination of liver tissues of PMC/Verapamil/Cimetidine group. After treatment with 300 mg/kg paracetamol, 150 mg/kg Verapamil and 150 mg/kg Cimetidine for two weeks, liver tissues with fixed with 10% formaldehyde, and proceed with H & E staining as described in Section 2.

The Group 5 was given daily I/P paracetamol in normal saline, I/P Cimetidine in distilled water and I/P Verapamil in normal saline daily for 2 weeks. The liver function tests were observed within normal limits with mean serum ALT of 37.01 ± 3.62 U/L, mean serum ALP of 28.37 ± 2.43 U/L, mean serum AST of 38.56 ± 1.94 U/L and mean serum Bilirubin of 0.13 ± 0.03 mg/dl. In this group, after light microscopy of H & E stained slides were classed to have normal histological structure (Figure 5).

### 3.2. Comparison within Groups

**Figure 6:**
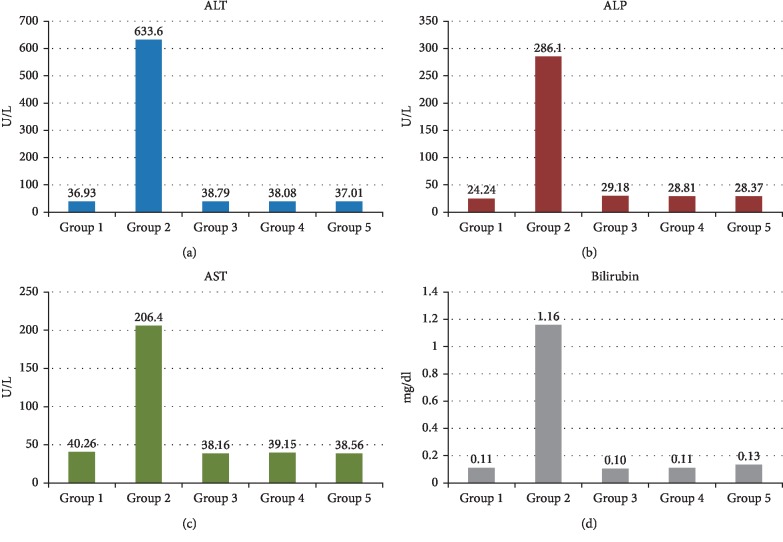
Comparison of mean levels of Liver function enzymes. (a) ALT (Alanine Aminotransferase) (b) ALP (Alkaline-Phosphatase enzyme) (c) AST (Aspartate Aminotransferase). (d) Bilirubin. Mean serum levels of all measured enzymes were combined to compare the level in various groups. *X*-axis represents the groups while enzymes level (U/L) are plotted on *Y*-axis.

Comparison of mean serum liver function parameters (Figure 6) between all the groups denoted a difference in the levels of ALT of Group 2 with *p* less than 0.05 in range.

## 4. Discussion

Fever, pain and inflammation are very commonly occurring issues in day to day life. A number of over the counter medications are available and used commonly for the relief of these symptoms. The use of paracetamol is very common. The hepatotoxicity induced by this drug does not present with any specific early symptoms and usually present with nausea along with vomiting that occurs a few hours after the intake of this drug in a dose that can produce toxicity. Pain in the abdomen soon pursues that persists for a period of 3-4 days [16]. The outcomes of the hepatic damage produced by paracetamol can be as severe as a fulminating failure of the liver and coma both of which have a bad prognosis [17]. There is rise in the level of the liver enzymes from the very beginning of the toxicity; however the maximum rise occurs within 3 days of the toxicity. About half of the patients with overdose of paracetamol present with failure of the liver, 20% of who require a liver transplantation.

The toxic metabolic product called NAPOI is formed and the stores of glutathione have depleted. So this product synthesized by the cytochromal enzymes leads to the liver damage, resulting in oxidative damage and mitochondrial dysfunction. One of the causes of the toxic liver injury can be an imbalance in the intracellular calcium homeostasis. The oxidative stress produced by the toxic metabolite of acetaminophen disturbs the intracellular calcium homeostasis which appears to play an important part in acetaminophen induced hepatotoxicity.

Keeping in mind the high incidence of use of paracetamol and its high propensity to produce hepatotoxicity, we decided to conduct a study with a view to investigate the hepatotoxic potential of paracetamol and its prevention by the use of Cimetidine and Verapamil, which can be used as hepatoprotective agents owing to their mechanism of action.. For this study BALB/C were chosen. These mice have certain characteristics which make them an animal model for studying hepatotoxicity. A study conducted by Bray in 1993, showed that BALB/C mice are very relevant models to human hepatotoxicity and can thus be used in studying drug induced hepatotoxicity [18]. Another study conducted by Muruganandan and Sinal, revealed that the reactive metabolic products of the drug bind to microsomes of the mice more frequently as compared to other animal models [19]. The parameters chosen for the assessment of liver damage in this study included enzyme ALT, AST, ALP as well as Bilirubin. The histopathology of the liver was done to look for the histopathological changes induced by paracetamol toxicity and to look for the protective effect of Cimetidine and Verapamil was performed. Cimetidine was selected for the study because it is an enzyme inhibitor and can be helpful in preventing the toxic effect of paracetamol. Verapamil is a blocker of calcium channel and it can prevent the hepatotoxicity of paracetamol occurring through calcium dependent mechanisms.

Group 1 was used as a control group that received normal food and water. The liver parameters assessed in the control group remained within normal limits. Histopathology was performed on the liver slides and it revealed normal architecture with no pathological findings. In Group 2 mice were subjected to daily intraperitoneal injection of 300 mg/kg Paracetamol. The liver parameters observed after 2 weeks showed a significantly raised levels of all the liver enzymes. The amount of rise of the ALT level was comparatively higher to the amount of rise in the ALP level. These results can be compared to a study performed by Metushi and colleagues [20]. Light microscopy of the specimen showed inflammatory changes with distorted tubular architecture. Group 3 received daily I/P injections of Paracetamol along with Cimetidine injections. All the assessed parameters were observed to be within normal limits. Liver histopathology revealed very mild histopathological changes. These findings can be compared to a study conducted by Rofe and colleagues [21]. Another study conducted by Juma and colleagues also showed similar findings [22]. Group 4 in the study was given daily I/P injections of paracetamol along with I/P Verapamil. The results of the tests revealed that all the parameters were within normal limits. There were mild to no histopathological changes observed. The hepatoprotective effect of Verapamil was also observed through a study conducted by Juma et al. [23]. Group 5 was given daily I/P injections of Paracetamol along with both Cimetidine as well as Verapamil. The assessment of the biochemical parameters following the co-administration of both Cimetidine and Verapamil showed an additional protective effect with all the parameters as well as the histopathology observed within normal limits.

Thus, it can be concluded that Cimetidine as well as Verapamil have a protective effect against paracetamol induced hepatotoxicity with similar efficacy. Further comparative studies among various commonly used hepato-protective agents should be conducted to investigate their protective potentials.

## 5. Conclusions

It can be concluded that paracetamol induced hepatotoxicity occurs through cytochromal enzymes as well as the imbalance in the calcium homeostasis. Cimetidine and Verapamil given together have an additive hepatoprotective effect in the paracetamol induced toxicity. Cimetidine reduces the hepatotoxic potential of paracetamol by reducing the formation of the toxic metabolite through cytochromal enzyme inhibition while Verapamil reduces the paracetamol induced hepatotoxicity by blocking the calcium channels thus, maintaining calcium balance and inhibiting the calcium dependent cellular damage.

## Figures and Tables

**Table 1:** Experimental designs followed in the study.

Groups (# of mice)	Paracetamol (PMC)	Cimetidine	Verapamil	Dissection
Group 1 (10)	—	—	—	Day 14
Group 2 (10)	300 mg/kg PMC daily	—	—	Day 14
Group 3 (10)	300 mg/kg PMC daily	150 mg/kg Cimetidine daily	—	Day 14
Group 4 (10)	300 mg/kg PMC daily	—	150 mg/kg Verapamil daily	Day 14
Group 5 (10)	300 mg/kg PMC daily	150 mg/kg Cimetidine daily	150 mg/kg Verapamil daily	Day 14

## Data Availability

The data used to support the findings of this study are included within the article; however the raw data files (images and enzyme kinetic assays) are available from the corresponding author upon request.
